# Hospital Admission Rate, Cumulative Hospitalized Days, and Time to Admission Among Older Persons With Substance Use and Psychiatric Conditions

**DOI:** 10.3389/fpsyt.2022.882542

**Published:** 2022-04-22

**Authors:** Wossenseged Birhane Jemberie, Mojgan Padyab, Dennis McCarty, Lena M. Lundgren

**Affiliations:** ^1^Department of Social Work, Umeå University, Umeå, Sweden; ^2^Centre for Demographic and Ageing Research (CEDAR), Umeå University, Umeå, Sweden; ^3^The Swedish National Graduate School on Aging and Health (SWEAH), Faculty of Medicine, Lund University, Lund, Sweden; ^4^School of Public Health, Oregon Health & Science University-Portland State University, Portland, OR, United States; ^5^Cross-National Behavioral Health Laboratory, Graduate School of Social Work, University of Denver, Denver, CO, United States

**Keywords:** aged, repeated hospitalizations, length of stay (D007902), older adult, dual diagnosis, substance use disorder, mental health disorders, comorbidities

## Abstract

**Background:**

Substance use among older persons occurs with medical and psychiatric comorbidities. This study examined the associations of substance use disorder (SUD), psychiatric, and dual diagnoses with 12-month cumulative hospitalized days, hospital admission rate and number of days to first hospitalization.

**Methods:**

The cohort of 3,624 individuals (28.2% women) aged 50 years or older was assessed for substance use severity in 65 Swedish municipalities during March 2003–May 2017. Addiction Severity Index data were linked to hospital discharge records and crime statistics. The outcomes were (a) 12-month cumulative hospitalized days; (b) Hospital admission rate, and (c) days to first hospitalization. Generalized linear regression techniques investigated associations between outcomes and SUD, psychiatric and dual diagnoses at admission.

**Results:**

During 2003–2017, 73.5% of the participants were hospitalized. Twelve-month hospitalized days were positively associated with SUD (Incidence rate ratio (IRR) = 1.41, 95%CI: 1.26–1.58), dual diagnosis (IRR = 2.03, 95%CI: 1.74–2.36), and psychiatric diagnoses (IRR = 2.51, 95%CI: 2.09–3.01). Hospital admission rate was positively associated with SUD (IRR = 4.67, 95%CI: 4.28–5.08), dual diagnosis (IRR = 1.83, 95%CI: 1.64–2.04), and psychiatric diagnoses (IRR = 1.73, 95%CI: 1.55–1.92). Days to first hospitalization were negatively associated with SUD (IRR = 0.52, 95%CI: 0.47–0.58), dual diagnosis (IRR = 0.57, 95%CI: 0.50–0.65), and psychiatric diagnoses (IRR = 0.83, 95%CI: 0.73–0.93). The marginal effects of SUD and/or mental disorders increased with age for all outcomes, except for days to first hospitalization.

**Conclusion:**

Three of four older persons assessed for substance use severity were later hospitalized. Substance use disorders, dual diagnoses and other mental disorders were the primary reasons for hospitalization and were associated with longer stays, earlier hospitalization, and repeated admissions. Sensitizing service providers to old age substance use and sharing data across the care continuum could provide multiple points of contact to reduce the risk of hospitalizations among older persons with problematic substance use.

## Introduction

Older persons using alcohol and other drugs, compared to younger people, are more susceptible to harms related to substance use due to age-related physiological changes and use of prescription drugs that may interact with alcohol and other drugs and lead to unplanned hospitalization (1–5). Detection of problematic substance use in older persons, however, can be challenging due to comorbid health conditions which mask effects of substance use; hence, practitioners may attribute symptoms to other health conditions and age-related physiological declines (6–8). Some clinicians are uncomfortable asking older patients about substance use (8–11). As a result, older persons might not be offered early intervention and treatment until coexisting health and psychosocial problems worsen.

Comorbid somatic and mental health disorders are common among older persons with chronic problematic substance use leading to resource-intensive old-age care, hospitalization, and post-discharge specialized care (7, 12–17). In Sweden, adults aged 50 years or older account for 66% of those hospitalized with alcohol use disorder and 20% with drug use disorder (18). The Swedish Association of Local Authorities and Regions (SALAR) estimates that one-third of psychiatric-related hospitalizations were among adults aged 65 years or older (19). More than 90% of inpatient stays due to psychiatric and SUD diagnoses among older patients, moreover, were unplanned and cost between US $3,000 and $6,300 (increasing cost with age) (19).

The Swedish substance use and mental health services are decentralized to regional and municipal services. The Social Services Act mandates that municipal social services provide *substance use assessments and psychosocial services* for treatment of problematic substance use (20, 21). The Health and Medical Services Act, however, assigns *the diagnosis of SUD and mental disorders* to the regional healthcare systems which provide low threshold services (such as needle exchange programs) and medical treatment for SUD, psychiatric and other comorbid conditions. The separate legal frameworks governing the social services, and the healthcare system complicate sharing patient data. Many older service users in Sweden have multiple hospital admissions prior to receiving an assessment for substance use severity and planned treatment services (12). Because of the fragmentation of substance use, mental and physical health services between municipal and regional authorities, those assessed for substance use severity may not receive the treatments they need leading to multiple unplanned hospitalizations. The Swedish government is investigating the transfer of substance use treatment from municipal social services to regional healthcare services (22). Knowledge about how often, how long and how early older persons are hospitalized after contact with municipal social services for assessment of substance use severity can inform the development of new policies for coordination of care among older persons.

This study examines the relationships between hospitalization and diagnoses of mental health and substance use disorders (at hospital admission) among older persons, aged 50+ years. An analysis linked multiple Swedish registries and assessed associations with 12-month cumulative hospitalized days, hospital admission rate and days to first hospital admissions following an assessment for substance use severity.

## Methods

### Study Setting and Data Sources

Most of Sweden’s substance use services are publicly funded and decentralized to regional and municipal services. The National Board of Health and Welfare (NBHW) recommends the use of Addiction Severity Index (ASI) for in-depth assessment of substance use problem severity and grading of treatment/intervention needs for substance use and related problems in other life domains (such as mental health and social problems). More than 90% of Swedish municipalities use the ASI to evaluate severity of substance use and related problems, and to plan need-based psychosocial treatments for adults with problematic substance use (23). Social workers with ASI training complete the assessments.

For this analysis, routinely collected municipal ASI assessment data were linked at the individual level to the Swedish Inpatient Register (hospital discharge records) from the NBHW and to the Swedish crime statistics data on persons found guilty of offences from the Swedish National Council for Crime Prevention (NCCP). The hospital discharge data were available from October 2000 to December 2017 and the crime statistics data from January 2002 to December 2017. The quality and coverage of the registers are reported previously (24–28).

### Study Design and Population

The retrospective cohort included all individuals aged 50 years or older (*n* = 3,624, 28.2% women) initially assessed for substance use severity by social services between March 2003 and May 2017, in 65 Swedish municipalities.

### Measurement

#### Dependent Variables

The analysis assessed three outcomes.

(A) *12-month Cumulative hospitalized days:* summing the number of days per individual for all-cause hospital admissions during the 12 months post ASI assessment.
(B) *Hospital admission rate:* the total number of post-ASI all-cause hospital admissions per person per follow-up time, until the end of the study period (March 2003 through December 2017).
(C) *Days to first hospital admission:* the number of days between ASI assessment and first hospital admission during the entire study period (March 2003 through December 2017).

#### Independent Variables

International Statistical Classification of Diseases, tenth revision (ICD-10) diagnostic codes were extracted from the national inpatient register. Three mutually exclusive diagnostic categories were used as binary independent variables (Absent/Present).

1. SUD-related diagnoses (ICD-10 codes *F10X-F19X* as principal or contributing cause of hospitalization with no other psychiatric comorbidity). Supplementary Table 1 lists the ICD-10 codes and frequency for disorders due to psychoactive substance use.
2. Psychiatric diagnoses (ICD-10 codes *F20-F29; F30-F39; F40-F48; F60-F62; F63-F69; F90-F99* as principal or contributing cause of hospitalization with no SUD comorbidity). Supplementary Table 2 lists the ICD-10 codes and frequency for different psychiatric diagnoses recorded during admission.
3. Dual diagnoses (defined in this study as hospital admissions with any SUD and other psychiatric diagnoses recorded during the same hospitalization episode regardless of their status as principal or secondary cause).

#### Covariates

Covariates were selected *a priori* based on previous studies on predictors of hospitalization and their availability in the data sources (29–35).

##### Sociodemographic Variables

Characteristics of the study sample extracted from the ASI register included: age (continuous variable, centered at 50 years), gender, country of birth, marital status, employment status, education, residence, town population size and housing status.

##### Criminal Justice Involvement

The NCCP crime statistics register provided data on involvement with the criminal justice system. A categorical variable (not sentenced; sentenced but not jailed; jailed at least once) was used to adjust for interrupted stay in community during the study periods as such interruption might reduce the likelihood of hospitalization.

##### Health-Related Variables

The national inpatient register provided data on hospitalization in the year prior to ASI interview (coded as yes vs. no). Physical comorbidities were coded as a categorical variable (0, 1, and 2+ comorbidities) using Quan and colleagues’ algorithm for Elixhauser’s comorbidity categories (36). To avoid multicollinearity, four comorbidity categories related to SUD, psychosis, and depression were excluded from the original 31 comorbidity categories. Supplementary Table 3 lists the ICD-10 codes used to generate the comorbidity categories.

### Statistical Analysis

Descriptive statistics compared participant characteristics of those who were hospitalized and were not hospitalized post-ASI assessment using Pearson’s χ*^2^* test for categorical variables and two-sample *t*-test for continuous variables. Generalized linear regression techniques were used because of overdispersion in the count dependent variables. A zero inflated negative binomial regression (ZINB) model examined the associations between 12-month cumulative hospitalized days and the independent variables. The analysis controlled for the number of hospital admission episodes during the 12 months post baseline assessment. For the analyses on post-ASI hospital admission rate and time to first hospital admission, a mean-dispersion negative binomial regression model accounted for the variation in exposure time from initial ASI assessment date (entry to the study) to the end date of the study period (December 2017). Average adjusted predicted values and marginal (partial) effects were calculated for the outcome and independent variables after the regression models were fitted. To investigate the differences in effects of independent variables that may exist across age, we calculated average adjusted predictions and marginal (partial) effects at representative values of age. Regression models also examined age based on five categories (50–54, 55–59, 60–64, 65–69, and ≥70 years old); see Supplementary Table 4 for the results. All statistical analyses were complete-case analyses and conducted using Stata Standard Edition version 15.1 (37).

### Ethical Consideration and Reporting Guideline

The Ethical Review Authority in Umeå, Sweden approved the study (DNR: 2016/504-31; amendment 2020-06233). The authority waived the need for patient consent and data were deidentified before they were released for analysis. The study followed the Strengthening the Reporting of Observational Studies in Epidemiology (STROBE) and its extension, the Reporting of Studies Conducted using Observational Routinely collected health Data (RECORD) reporting guidelines (38, 39). See Supplementary Table 5 for the *STROBE* and *RECORD* statement checklists.

## Results

Between 2003 and 2017, 2,664 of 3,624 (73.5%) individuals were hospitalized one or more times following an index ASI assessment for substance use severity. See Table 1 for the characteristics of the cohort. About 54.5% (*n* = 1,976) were admitted with SUD diagnoses, 10.9% (*n* = 395) with dual diagnoses and 12.0% (*n* = 434) with psychiatric diagnoses. Furthermore, 14.2% (*n* = 514) were hospitalized with other diagnoses. Those who were hospitalized, compared to those not hospitalized, were older, not married, had lower employment rates, less education, and more history of hospitalization (see Table 1).

**TABLE 1:** Baseline characteristics of the study population, those who were not hospitalized and those who were hospitalized one or more time between March 2003 and December 2017.

	Total	Never hospitalized	Hospitalized during 2003–2017	*p*-value

	***N* = 3,624**	***N* = 960**	***N* = 2,664**	
*Age, mean (SD)*	57.30 (5.71) (*n* = 3,624)	56.96 (5.83) (*n* = 960)	57.42 (5.66) (*n* = 2,664)	0.032
*Age Category*				0.005
50–54 years old	1,401 (38.7%)	411 (42.8%)	990 (37.2%)	
55–59 years	1,086 (30.0%)	277 (28.9%)	809 (30.4%)	
60–64 years	692 (19.1%)	157 (16.4%)	535 (20.1%)	
65–69 years	324 (8.9%)	76 (7.9%)	248 (9.3%)	
70–83 years	121 (3.3%)	39 (4.1%)	82 (3.1%)	
*Gender*				0.11
Man	2,599 (71.7%)	669 (69.7%)	1,930 (72.4%)	
Woman	1,023 (28.2%)	290 (30.2%)	733 (27.5%)	
Missing	2 (0.1%)	1 (0.1%)	1 (<1%)	
*Country of birth*				0.46
Born in Sweden; Swedish parents	2,745 (75.7%)	714 (74.4%)	2,031 (76.2%)	
Born in other Nordic countries	329 (9.1%)	85 (8.9%)	244 (9.2%)	
Born outside of Sweden and Nordic countries	176 (4.9%)	56 (5.8%)	120 (4.5%)	
Born in Sweden: Nordic parents	196 (5.4%)	56 (5.8%)	140 (5.3%)	
Born in Sweden: non-Nordic parents	116 (3.2%)	33 (3.4%)	83 (3.1%)	
Missing	62 (1.7%)	16 (1.7%)	46 (1.7%)	
*Marital status*				0.002
Married/cohabiting	979 (27.0%)	300 (31.3%)	679 (25.5%)	
Separated/widowed	2,264 (62.5%)	560 (58.3%)	1,704 (64.0%)	
Never married/cohabited	264 (7.3%)	68 (7.1%)	196 (7.4%)	
Missing	117 (3.2%)	32 (3.3%)	85 (3.2%)	
*Usual Employment pattern, past 3 years*				<0.001
Full/part time employed	1,360 (37.5%)	428 (44.6%)	932 (35.0%)	
unemployed/irregular/disability	1,774 (49.0%)	418 (43.5%)	1,356 (50.9%)	
pension for retired	363 (10.0%)	85 (8.9%)	278 (10.4%)	
other: study/conscripted/institutionalized	33 (0.9%)	9 (0.9%)	24 (0.9%)	
Missing	94 (2.6%)	20 (2.1%)	74 (2.8%)	
*Education level*				<0.001
<9 years	514 (14.2%)	98 (10.2%)	416 (15.6%)	
Between 9 and 12 years	1,600 (44.2%)	430 (44.8%)	1,170 (43.9%)	
Completed 12 years	448 (12.4%)	117 (12.2%)	331 (12.4%)	
>12 years	953 (26.3%)	280 (29.2%)	673 (25.3%)	
Missing	109 (3.0%)	35 (3.6%)	74 (2.8%)	
*Past year hospitalization history: pre ASI interview*				<0.001
Not hospitalized	1,680 (46.4%)	656 (68.3%)	1,024 (38.4%)	
Hospitalized 1 or more times	1,944 (53.6%)	304 (31.7%)	1,640 (61.6%)	
*Criminal justice involvement*				0.14
Not sentenced the 12 m pre-ASI assessment	3,230 (89.1%)	869 (90.5%)	2,361 (88.6%)	
Sentenced, not jailed the 12 m pre-ASI assessment	270 (7.5%)	67 (7.0%)	203 (7.6%)	
Jailed at least once in the 12 m pre-ASI assessment	124 (3.4%)	24 (2.5%)	100 (3.8%)	
*Residential town population size*				0.21
Big-size town > 100,000 pop.	1,682 (46.4%)	423 (44.1%)	1,259 (47.3%)	
medium-size town 10–100,000 pop	1,612 (44.5%)	446 (46.5%)	1,166 (43.8%)	
Small-size town < 10,000 pop	305 (8.4%)	86 (9.0%)	219 (8.2%)	
Missing	25 (0.7%)	5 (0.5%)	20 (0.8%)	
*Housing condition*				0.30
Stable housing	2,693 (74.3%)	728 (75.8%)	1,965 (73.8%)	
Living with others permanently	209 (5.8%)	42 (4.4%)	167 (6.3%)	
Provided by social services	251 (6.9%)	65 (6.8%)	186 (7.0%)	
Unstable housing/Homeless	302 (8.3%)	81 (8.4%)	221 (8.3%)	
Other e.g., hotel	140 (3.9%)	37 (3.9%)	103 (3.9%)	
Missing	29 (0.8%)	7 (0.7%)	22 (0.8%)	

*Pairwise comparisons were based on non-missing data. p-values were calculated based on two-sample t-test for the variable “age” and Pearson’s χ^2^ test for categorical variables, between those never hospitalized and those hospitalized at least once.*

When limiting the analysis to the 12 months post ASI assessment, two in five participants (42.7%; *n* = 1,549) were hospitalized at least once during the 12 months post ASI assessment. Of those, 73% (*n* = 1,130) were hospitalized with SUD, 10.8% (*n* = 168) with dual diagnoses and 7.4% (*n* = 115) with other psychiatric diagnoses. About 20% (*n* = 310) were admitted with diagnoses other than psychiatric and/or SUD conditions.

### Twelve-Month Cumulative Hospitalized Days (Inpatient Days)

The mean number of days spent in hospital during the 12 months following the ASI index assessment was 9.9 with a positive coefficient of skewness of 4.9 suggesting over-dispersion. See Table 2 for the incidence rate ratio (IRR) for the independent variables and the covariates included in the analysis for 12-month cumulative hospitalized days (second column, Table 2). The average adjusted predictions and average marginal effects for the independent variables are reported in Table 3.

**TABLE 2:** Incidence rate ratios (IRR) for substance use disorders (SUD), dual and psychiatric diagnoses associated with 12-month hospitalized days, hospital admission rate and time to hospital admission.

Variables	12-months hospitalized days	Hospital admission rate	Time to admission
Independent Variables	IRR (95% CI)	IRR (95% CI)	IRR (95% CI)
*Types of diagnoses*			
SUD diagnoses: No	1 (reference)	1 (reference)	1 (reference)
SUD diagnoses: Yes	**1.41 (1.26**–**1.58)**	**4.67 (4.28**–**5.08)**	**0.52 (0.47**–**0.58)**
Dual diagnoses: No	1 (reference)	1 (reference)	1 (reference)
Dual diagnoses: Yes	**2.03 (1.74**–**2.36)**	**1.83 (1.64**–**2.04)**	**0.57 (0.50**–**0.65)**
Psychiatric diagnoses: No	1 (reference)	1 (reference)	1 (reference)
Psychiatric diagnoses: Yes	**2.51 (2.09**–**3.01)**	**1.73 (1.55**–**1.92)**	**0.83 (0.73**–**0.93)**
**Covariates**			
*Past year hospitalization history: before ASI interview*			
Not hospitalized	1 (reference)	1 (reference)	1 (reference)
Hospitalized 1 or more times	**1.32 (1.18**–**1.47)**	**1.77 (1.63**–**1.91)**	**0.67 (0.62**–**0.74)**
*Physical comorbidity*			
0 Elixhauser physical comorbidity	1 (reference)	1 (reference)	1 (reference)
1 Elixhauser physical comorbidity	**1.38 (1.23**–**1.56)**	**1.65 (1.50**–**1.80)**	**0.77 (0.70**–**0.86)**
≥2 Elixhauser physical comorbidities	**1.62 (1.44**–**1.82)**	**2.18 (1.99**–**2.39)**	**0.68 (0.62**–**0.76)**
*Age (continuous measure)*	**1.01 (1.00**–**1.02)**	**1.02 (1.01**–**1.03)**	1.00 (0.99–1.01)
*Gender*			
Man	1 (reference)	1 (reference)	1 (reference)
Woman	**0.84 (0.76**–**0.94)**	0.98 (0.90–1.07)	1.07 (0.97–1.18)
*Country of birth*			
Born in Sweden: Swedish parents	1 (reference)	1 (reference)	1 (reference)
Born in other Nordic countries	0.88 (0.75–1.03)	1.01 (0.89–1.15)	**0.83 (0.71**–**0.96)**
Born outside of Sweden and Nordic Countries	0.91 (0.72–1.15)	0.95 (0.79–1.14)	0.99 (0.80–1.21)
Born in Sweden: Nordic parents	0.87 (0.69–1.09)	0.90 (0.75–1.06)	1.12 (0.93–1.36)
Born in Sweden: non-Nordic parents	0.92 (0.70–1.20)	**1.37 (1.11**–**1.69)**	0.91 (0.71–1.17)
*Marital status*			
Married/cohabiting	1 (reference)	1 (reference)	1 (reference)
Separated/widowed	0.98 (0.88–1.11)	1.05 (0.96–1.15)	0.97 (0.88–1.08)
Never married/cohabited	**1.31 (1.07**–**1.61)**	**1.22 (1.05**–**1.43)**	0.99 (0.82–1.18)
*Usual employment pattern, past 3 years*			
Full/part time employed	1 (reference)	1 (reference)	1 (reference)
Unemployed/irregular/disability	1.06 (0.95–1.18)	1.04 (0.96–1.13)	0.96 (0.87–1.06)
Pension for retired	1.10 (0.91–1.35)	1.02 (0.87–1.20)	0.92 (0.77–1.11)
Study/conscripted/institutionalized	1.16 (0.67–2.01)	1.23 (0.84–1.78)	0.87 (0.55–1. 37)
*Education level*			
Less than 9 years	1 (reference)	1 (reference)	1 (reference)
Between 9 and 12 years	0.96 (0.83–1.10)	**1.15 (1.03**–**1.29)**	1.11 (0. 98–1. 27)
Completed 12 years	1.09 (0.91–1.30)	**1.22 (1.06**–**1.41)**	1.00 (0.85–1.17)
More than 12 years	**1.24 (1.06**–**1.45)**	**1.24 (1.10**–**1.40)**	1.08 (0.94–1.25)
*Residential town population size*			
<10,000	1 (reference)	1 (reference)	1 (reference)
Between 10,000 and 100,000	1.06 (0.88–1.28)	**1.22 (1.06**–**1.40)**	0.98 (0.83–1.15)
>100,000	1.07 (0.89–1.28)	**1.31 (1.14**–**1.50)**	0.96 (0.82–1.13)
*Housing condition*			
Stable housing	1 (reference)	1 (reference)	1 (reference)
Living with others permanently	**1.23 (1.01**–**1.51)**	1.09 (0.94–1.28)	1.07 (0.90–1.28)
Housing provided by social services	0.92 (0.76–1.12)	0.98 (0.84–1.13)	**1.19 (1.00**–**1.41)**
Unstable housing/homeless	**1.23 (1.03**–**1.47)**	0.99 (0.87–1.14)	1.06 (0.90–1.25)
Other e.g., hotel	0.91 (0.70–1.18)	1.03 (0.85–1.24)	1.21 (0.97–1.52)
*Criminal justice involvement*			
Not sentenced post ASI assessment	1 (reference)	1 (reference)	1 (reference)
Sentenced, but not jailed post ASI assessment	1.00 (0.84–1.19)	1.10 (0.99–1.21)	1.02 (0.90–1.15)
Jailed 1 or more time post ASI assessment	1.16 (0.89–1.52)	**1.16 (1.00**–**1.34)**	1.04 (0.88–1.23)
*Intercept*	**5.26 (4.04**–**6.85)**	**0.00 (0.00**–**0.00)**	**0.55 (0.44**–**0.70)**
alpha	**0.74 (0.69**–**0.80)**	**0.70 (0.66**–**0.75)**	**1.15 (1.09**–**1.21)**
*N*	3,391	3,378	2,494

*Bold values indicate p-value < 0.05.*

**TABLE 3:** Average adjusted predictions and average marginal effects for substance use disorders (SUD), dual and psychiatric diagnoses.

Variables	12-months hospitalized days	Hospital admission rate	Time to admission
*Types of diagnoses*			
SUD diagnoses: No	5.53	2.08	863.53
SUD diagnoses: Yes	7.81	9.68	452.36
*AME (95% CI): Yes vs. No*	***2.28 (1.57*–*2.99)***	***7.61 (7.13*–*8.08)***	***–411.17 (–487.69* to *–334.65)***
Dual diagnoses: No	6.38	6.46	603.23
Dual diagnoses: Y es	12.91	11.83	343.40
*AME (95% CI): Yes vs. No*	***6.54 (4.66***–***8.41)***	***5.37 (4.16***–***6.57)***	***–259.82 (–308.38* to *–211.27)***
Psychiatric diagnoses: No	6.42	6.54	588.45
Psychiatric diagnoses: Yes	16.12	11.30	487.35
*AME (95% CI): Yes vs. No*	***9.70 (6.86***–***12.54)***	***4.76 (3.63***–***5.89)***	***–101.10 (–161.79* to *–40.42)***

*AME, Average Marginal Effect. Bold values indicate AMEs are significantly different from zero.*

The total number of hospitalized days over the 12 months post ASI assessment was positively associated with psychiatric diagnoses (IRR = 2.51, 95% CI: 2.09–3.01), dual diagnoses (IRR = 2.03, 95% CI: 1.74–2.36) and SUD-related diagnoses (IRR = 1.41, 95% CI: 1.26–1.58) (Table 2). The corresponding increase in inpatient stays, keeping all other variables at their observed values, were 9.7 days for psychiatric diagnoses, 6.5 days for dual diagnoses and 2.3 days for SUD-related diagnoses (Table 3). Other covariates positively associated with number of days hospitalized included history of hospitalization before the index ASI assessment, having a physical comorbidity, increasing age, male, never married, more than 12 years of education, unstable housing condition and living with others permanently.

### Hospital Admission Rate

The cohort had a mean of 6.4 hospital admissions following the ASI assessment with a 4.3 positive coefficient of skewness suggesting overdispersion. After accounting for exposure time, SUD-related diagnoses (IRR = 4.67, 95% CI: 4.28–5.08), dual diagnoses (IRR = 1.83, 95% CI: 1.64–2.04) and other psychiatric diagnoses (IRR = 1.73, 95% CI: 1.55–1.92) (Table 2) were positively associated with hospital admission rate. The average increase in hospital admission rate was 7.61 per person-year for SUD diagnoses, 5.37 for dual diagnoses and 4.76 for psychiatric diagnoses while holding other covariates at their observed values (Table 3). Higher admission rate was also associated with prior hospitalizations, more physical comorbidities, aging, being born in Sweden with parents from non-Nordic countries, never married, more education, living in bigger towns, and being incarcerated during the study period (Table 2).

### Time From Initial Addiction Severity Index Interview to First Hospital Admission

The mean number of days to first hospitalization among those who were hospitalized was 531.2 days with standard deviation of 658.7. The number of days from index ASI intake to first hospitalization among those with SUD-related diagnoses was 411.2 days less than for those without a SUD diagnosis (IRR = 0.52, 95% CI: 0.47–0.58). Individuals with a dual diagnosis were hospitalized 259.8 days earlier than those without a dual diagnosis (IRR = 0.57, 95% CI: 0.50–0.65). The time to hospitalization among those with a psychiatric diagnosis was 101.1 days earlier when compared to those without a psychiatric diagnosis (IRR = 0.83, 95% CI: 0.73–0.93). Prior hospitalization history, having physical comorbidities, and being born in Nordic countries other than Sweden were associated with earlier hospital admissions following ASI assessment. Receipt of housing support provided by public social services was associated with more days to hospitalization.

### Average Marginal Effects of Substance Use Disorder and/or Mental Disorders Across Age

**FIGURE 1:**
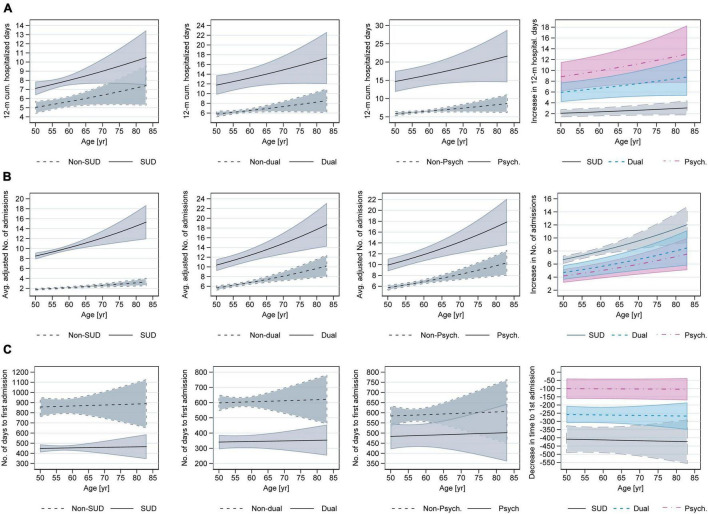
Adjusted predictions and partial effects for SUD, dual and psychiatric diagnoses across age given for 3 different outcomes: 12-month cumulative hospitalized days **(A)**; Hospital admission rate **(B)**; Time to first hospital admission **(C)**. The last columns in the figure show partial effects of the independent variables across age. For example, the partial effect for SUD is calculated as the change from no SUD diagnoses to SUD diagnoses. The shaded area in the graphs show 95% confidence intervals.

The partial effects of SUD, psychiatric and dual diagnoses on the hospitalization rate and 12-month cumulative inpatient days increased with age while their effect on the time to first hospitalization did not vary by age (Figure 1).

**FIGURE 2:**
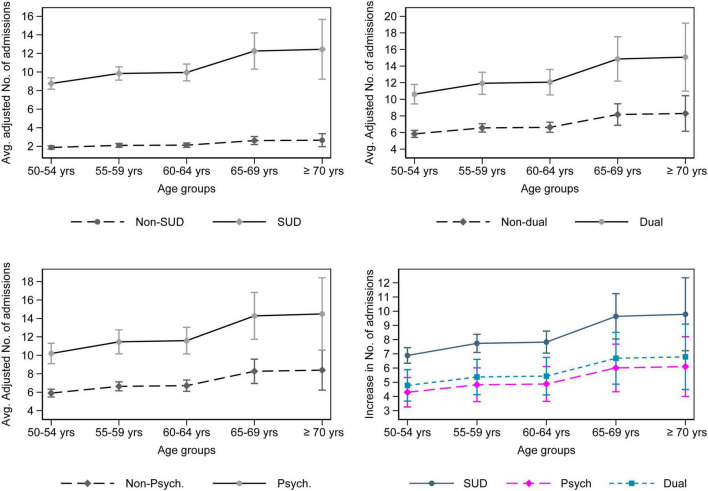
Adjusted predictions and partial effects of SUD, dual and psychiatric diagnoses across five age groups for hospital admission rate. The figure on the bottom right depicts the partial effects for the diagnoses vs. not having the respective diagnoses.

The analyses with five age categories found that only the association with hospital admission rate remained significant (see Supplementary Table 4). Compared to 50–54 years group, older age groups had higher incidence rate ratios. For 55–59 years the IRR was 1.12 (95% CI: 1.03–1.23); for 60–64 years IRR = 1.14 (95% CI: 1.02–1.26); for 65–69 years IRR = 1.40 (95% CI: 1.17–1.67) and for those older than 70 years old, the IRR was 1.42 (95% CI: 1.08–1.87). The partial effects for SUD, dual and psychiatric diagnoses increased for the 55–59 years group when compared to 50–54 years group. The effects, however, plateaued for 60–64 years group before increasing again for age group 65–69 years and plateaued again for those older than 70 years (Figure 2).

## Discussion

The retrospective cohort study of older persons using alcohol and other drugs investigated the association of SUD, psychiatric and dual diagnoses with three outcome measures: 12-month cumulative hospitalized days, hospital admission rate, and time to first hospitalization. During the study period, almost three-fourth were hospitalized following their initial ASI assessment at municipal social services. More than half were hospitalized with SUD and about one-fifth with either non-SUD related psychiatric conditions or dual diagnosis. While the analysis did not consider what type of treatments service users were provided, the high proportion of hospitalization implies that for some their substance use related treatment needs might not have been met. These findings aligned with previous reports and suggested that medical and psychiatric comorbidities are prevalent among older persons with substance use problem leading to increased healthcare utilization (13, 15, 40–42).

While some older persons assessed for substance use severity might not fulfill the SUD diagnosis criteria, previous studies suggest that using even a small quantity of substance might harm older persons especially those with comorbidities and complex care needs (13, 43, 44). Additionally, co-occurrence of psychiatric diagnosis among older persons with substance use disorder is associated with medical multimorbidity, lower medication adherence, severe medication interactions, repeated hospital admission and longer hospital stay (13, 43, 45–47). Our analysis suggests SUD, psychiatric and dual diagnoses are associated with longer cumulative hospitalized days, higher hospitalization rate, and shorter number of days between initial ASI assessment and first hospitalization. For cumulative hospitalized days and hospital admission rate, the partial effects of SUD and/or psychiatric diagnoses increased with age supporting previous studies which found those aging with problematic substance use have higher healthcare utilization (13, 16, 41, 48, 49). For those who were hospitalized following ASI assessment, however, the partial effects of SUD and/or psychiatric diagnoses on the number of days to first hospitalization did not vary across age. This might be explained by the fact that the analysis included only those hospitalized, who were older and more likely to have a prior inpatient stay than those not hospitalized.

While the current study does not investigate the effect of contextual factors such as coordination of care for substance use and mental health, analysis from *The Swedish Agency for Health and Care Services Analysis* reported that the majority of service users were not satisfied with the coordination of their treatments within health and social services, and many were forced to coordinate their own treatments between service providers (50). Moreover, official data suggests that more than 90% of hospital admissions due to psychiatric and SUD diagnoses among older patients are unplanned (19). The current study found that most older persons assessed for substance use severity were later hospitalized with SUD and/or psychiatric diagnoses.

In addition to SUD treatment, municipal social services also provide old-age care. While this double responsibility theoretically makes social services ideal treatment providers for older persons with substance use problem, almost three-fifth of the municipalities in Sweden lack any coordination strategy between old-age care and substance use services, and only 11% of municipal old-age care services use standardized instrument to screen for SUD (17, 51). Those providing old-age care either lack the competence or the resources to identify and treat substance use disorder among older service users (52, 53). Moreover, only 40% of municipalities have joint agreements with regional entities to coordinate social and health care services (51) despite legislation stipulating the need for a coordinated individual plan for those in need of care from both social services and healthcare services such as those with dual diagnoses (50). The Swedish government is investigating the transfer of addiction treatment from social services to healthcare services (21, 22). Integrating substance use and mental health services into primary care, training multidisciplinary care providers on old-age problematic substance use, streamlining data sharing within and between health and social care services could result in earlier identification and treatment of substance use disorder and comorbid conditions among older persons (6, 45, 54–59).

### Strengths and Limitations

The linkage of routinely collected data enabled us to include relevant variables into the analysis. We cannot, however, rule out the possibility of unmeasured treatment-related and contextual-level cofounders. Some individuals, for example, might have accessed outpatient specialty addiction and mental health clinics or residential addiction care which could alter the likelihood of hospitalization post ASI-assessment.

The social workers who conduct substance use severity assessments are trained on using ASI which increases the quality of collected data. The National Board of Health and Welfare recommends the use of ASI for both initial assessment and six-month follow-up interview with service users (using “ASI-baseline” and “ASI follow-up” instruments, respectively). While ASI-baseline is used by almost 93% of the municipalities, the use of “ASI follow-up” post-treatment is very limited. Moreover, we did not have data on the treatments provided to the service users post their ASI assessment. Receipt of or completion of a treatment service was not, however, a variable considered in the current study.

The authorities maintaining the registries used in the current study conduct routine quality controls. While all three registers have high quality and coverage, use of data which were not collected for research purposes entails limitations such as misclassification of cases, lag in data entry, missingness of data and change in variable operationalization. In this study, missingness of data was generally modest.

Another limitation of the study is that due to few hospitalizations across some diagnoses, we did not consider fine-grain ICD-10 codes in our analysis to investigate if the outcome variables varied across different mental health and substance use disorders.

A final limitation notes that the ASI data were collected from the municipal social services which provide most of substance use services. Many older persons with problematic substance use, however, refrain from using these services due to stigma until the problem worsens and a need for financial and housing support arises (20). Some older persons might also seek medical treatment for SUD from healthcare services. The fact that in Sweden, SUD treatment is publicly funded might limit the generalizability of our findings for a setting where treatment services for SUD and comorbid conditions are funded and organized differently.

## Conclusion

Almost 75% of older persons who were assessed for substance use severity were later hospitalized due to any cause. Substance use disorders, dual diagnosis and other mental disorders were the main reasons for hospitalization and were all associated with longer 12-month cumulative hospitalized days, higher admission rate and shorter time to hospitalization. These effects, except for time to hospitalization, become even more pronounced with increasing age. Older persons often have multiple care needs from social services, primary care, and specialist healthcare services. Sensitizing service providers to late life substance use and sharing data across the care continuum could provide multiple points of contact to early identify and to provide timely and equitable care and to reduce the risk of hospitalizations among older persons with substance use problem.

## Data Availability Statement

The datasets presented in this article are not readily available because of legal and ethical restrictions. Codes for data preparation and analyses, and Stata log files can be shared through correspondence with WJ (wossenseged.jemberie@umu.se). ASI data are available *via* the Swedish municipalities. Hospital discharge data are available *via* the National Board of Health and Welfare. Crime statistics data on persons found guilty of offences are available *via* the Swedish National Council for Crime Prevention. Requests to access the datasets should be directed to Socialstyrelsen, patientregistret@socialstyrelsen.se; Brottsförebyggande rådet, statistik@bra.se.

## Ethics Statement

The studies involving human participants were reviewed and approved by the Ethical Review Authority in Umeå, Sweden (DNR: 2016/504-31; amendment 2020-06233). Written informed consent for participation was not required for this study in accordance with the national legislation and the institutional requirements.

## Author Contributions

WJ: conceptualization (lead), methodology (lead), formal analysis (lead), writing—original draft (lead), writing—review and editing (lead), visualization (lead), and funding acquisition (equal). MP: conceptualization (supporting), writing—review and editing (supporting), supervision (lead), validation (equal), and project administration (supporting). DM: conceptualization (supporting), writing—review and editing (equal), supervision (supporting), and validation (equal). LL: funding acquisition (lead), project administration (lead), writing—review and editing (supporting), validation (equal), and supervision (supporting). All authors reviewed and approved submission of the manuscript.

## Conflict of Interest

The authors declare that the research was conducted in the absence of any commercial or financial relationships that could be construed as a potential conflict of interest.

## Publisher’s Note

All claims expressed in this article are solely those of the authors and do not necessarily represent those of their affiliated organizations, or those of the publisher, the editors and the reviewers. Any product that may be evaluated in this article, or claim that may be made by its manufacturer, is not guaranteed or endorsed by the publisher.
