# Supplementary material for: Hospital Admission Rate, Cumulative Hospitalized Days, and Time to Admission Among Older Persons With Substance Use and Psychiatric Conditions
Source: Front Psychiatry. 2022 Apr 22;13:882542. doi: 10.3389/fpsyt.2022.882542 (PMC9075517; doi:10.3389/fpsyt.2022.882542)
Supplement: Supplementary file 1 [file Data_Sheet_1.pdf]

Hospital Admission Rate, Cumulative Hospitalized Days, and Time to Admission Among Older Persons with Substance Use and Psychiatric Conditions

Authors' List, Order and Affiliation

Wossenseged Birhane Jemberie, MPH. [1, 2, 3*]
PhD candidate. **1.** Department of Social Work, Umeå University, Umeå, Sweden;
2. Centre for Demographic and Ageing Research (CEDAR), Umeå University, Umeå, Sweden;
3. The Swedish National Graduate School on Aging and Health (SWEAH), Faculty of Medicine, Lund University, Sweden. E-mail: wossenseged.jemberie@umu.se

Mojgan Padyab, PhD. [1, 2]
Associate professor. **1.** Department of Social Work, Umeå University, Umeå, Sweden;
2. Centre for Demographic and Ageing Research (CEDAR), Umeå University, Umeå, Sweden. Email: mojgan.padyab@umu.se

Dennis McCarty, PhD. [4]
Professor Emeritus. **4.** Oregon Health & Science University- Portland State University, School of Public Health, Portland, Oregon, United States. Email: mccartyd@ohsu.edu

Lena M. Lundgren, PhD. [1, 5]
Professor. **1**. Department of Social Work, Umeå University, Umeå, Sweden;
5. Cross-National Behavioral Health Laboratory, Graduate School of Social Work, University of Denver, Denver, Colorado, United States. Email: lena.lundgren@du.edu

***Send correspondence to** Wossenseged Birhane Jemberie. Department of Social Work, Umeå University, 901 87 Umeå, Sweden. E-mail: wossenseged.jemberie@umu.se

Running Head*: Old-age Substance Use and Hospitalization*

Supplementary Table 1. Number of individuals and number of admissions with substance use disorders as principal or underlying cause of hospital admission during 2003-2017 following ASI assessment

Mental and behavioural disorders due to use of:	ICD-10 codes	Principal diagnosis	Underlying cause of admission
		**Unique individuals (total no. of episodes)**	**Unique individuals (total no. of episodes)**
alcohol	F10	1452 (10520)	1522 (7182)
opioids	F11	73 (171)	67 (143)
cannabinoids	F12	8 (9)	22 (31)
sedatives or hypnotics	F13	53 (98)	84 (144)
cocaine	F14	2 (2)	4 (4)
other stimulants, including caffeine	F15	48 (81)	64 (129)
hallucinogens	F16	1 (1)	3 (3)
tobacco	F17	0	212 (337)
volatile solvents	F18	0	2 (2)
multiple drug use and use of other psychoactive substances	F19	196 (529)	203 (505)

Note: Individuals with multiple hospitalizations can be admitted with different diagnoses during each episode. ASI = Addiction Severity Index; ICD-10 = International Statistical Classification of Diseases, tenth revision

Supplementary Table 2. Number of individuals and number of admissions with mental disorders other than substance use disorders as principal or underlying cause of hospital admission during 2003-2017 following ASI assessment

**Mental disorders (Psychiatric Conditions)**	**ICD-10 codes**	**Principal diagnosis**	**Underlying cause of admission**
		**Unique individuals (total no. of episodes)**	**Unique individuals (total no. of episodes)**
Schizophrenia Spectrum and other Psychotic Disorders	F20-F29 (excl. F21)	41 (81)	42 (118)
Personality Disorders	F21; F60-F62	19 (33)	38 (106)
Manic disorder	F30	6 (8)	3 (3)
Bipolar Disorder	F31	49 (128)	82 (268)
Depressive Disorders	F32-F33; F34.1	144 (294)	267 (503)
Persistent mood disorder	F34 (excl. F34.0 & F34.1)	0	2 (7)
Other mood disorder	F38	0	1 (1)
Unspecified mood disorder	F39	0	13 (15)
Anxiety Disorders	F40-F41 (excl. F41.2)	77 (158)	225 (497)
mixed anxiety-depressive disorder	F41.2	56 (116)	123 (206)
Obsessive Compulsive Disorder	F42; F45.2; F63.3	2 (4)	12 (22)
Trauma and Stressor-Related Disorders	F43; F94.1; F94.2	69 (94)	56 (81)
Dissociative Disorders	F44	1 (8)	4 (18)
Somatic Symptom and Related Disorders	F45 (excl. F45.2)	2 (3)	4 (5)
Neurasthenia disorders	F48	0	2 (2)
Disruptive, Impulse Control and Conduct Disorders	F63 (excl. F63.3); F91	0	4 (4)
Attention-deficit hyperactivity disorder (Hyperkinetic disorders)	F90	11 (20)	86 (283)
Miscellaneous mental disorders	Remaining F-codes excluding F00-F09 & substance use disorders	71 (89)	52 (99)

Note: Individuals with multiple hospitalizations can be admitted with different diagnoses during each episode. ASI = Addiction Severity Index; ICD-10 = International Statistical Classification of Diseases, tenth revision

Supplementary Table 3. Modified Elixhauser Comorbidity Categories and list of ICD-10 codes

**Category**	**ICD-10 codes**
Congestive heart failure	I099, I110, I130, I132, I255, I420, I425, I426, I427, I428, I429, I43, I50, P290
Cardiac arrhythmia	I441, I442, I443, I456, I459, I47, I48, I49, R000, R001, R008, T821, Z450, Z950
Valvular disease	A520, I05, I06, I07, I08, I091, I098, I34, I35, I36, I37, I38, I39, Q230, Q231, Q232, Q233, Z952, Z953, Z954
Pulmonary circulation disorders	I26, I27, I280, I288, I289
Peripheral vascular disorders	I70, I71, I731, I738, I739, I771, I790, I792, K551, K558, K559, Z958, Z959
Hypertension uncomplicated	I10
Hypertension complicated	I11, I12, I13, I15
Paralysis	G041, G114, G801, G802, G81, G82, G830, G831, G832, G833, G834, G839
Other neurological disorders	G10, G11, G12, G13, G20, G21, G22, G254, G255, G312, G318, G319, G32, G35, G36, G37, G40, G41, G931, G934, R470, R56
Chronic pulmonary disease	I278, I279, J40, J41, J43, J44, J45, J46, J47, J60, J61, J62, J63, J64, J65, J66, J67, J684, J701, J703
Diabetes uncomplicated	E100, E101, E109, E110, E111, E119, E120, E121, E129, E130, E131, E139, E140, E141, E149
Diabetes complicated	E102-E108, E112-E118, E122-E128, E132-E138, E142-E148
Hypothyroidism	E00-E03, E890
Renal Failure	I120, I131, N18, N19, N250, Z490, Z491, Z492, Z940, Z992
Liver disease	B18, I85, I864, I982, K70, K711, K713, K714, K715, K717, K72, K73, K74, K760, K762-K769, Z944
Peptic ulcer excluding bleeding	K257, K259, K267, K269, K277, K279, K287, K289
HIV/AIDS	B20, B21, B22, B24
Lymphoma	C81-C85, C88, C96, C900, C902
Metastic cancer	C77-C80
Solid tumour without metastasis	C00-C26, C30-C34, C37-C41, C43, C45-C58, C60-C76, C97
Rheumatoid arthritis/collagen	L940, L941, L943, M05, M06, M08, M120, M123, M30, M310-M313, M32, M33, M34, M35, M45, M461, M468, M469
Coagulopathy	D65, D66, D67, D68, D691, D693, D694, D695, D696
Obesity	E66
Weight loss	E40, E41, E42, E43, E44, E45, E46, R634, R64
Fluid and electrolyte disorders	E222, E86, E87
Blood loss anaemia	D500
Deficiency anaemia	D508, D509, D51, D52, D53

Note: ICD-10 = International Statistical Classification of Diseases, tenth revision

Supplementary Table 4. Incidence Rate Ratios (IRR) for variables affecting 12-month hospitalized days, hospital admission rate and time to hospital admission: Results from analysis with 5 age categories

Variables	12-months Hospitalized days	Hospital Admission Rate	Time to admission
**Variables**	**IRR (95% CI)**	**IRR (95% CI)**	**IRR (95% CI)**
Types of diagnoses			
SUD diagnoses: No	1 (reference)	1 (reference)	1 (reference)
SUD diagnoses: Yes	**1.41 (1.26 – 1.58)**	**4.68 (4.29 - 5.10)**	**0.52 (0.47 - 0.58)**
Dual diagnoses: No	1 (reference)	1 (reference)	1 (reference)
Dual diagnoses: Yes	**2.03 (1.74 – 2.36)**	**1.82 (1.63 - 2.03)**	**0.57 (0.50 - 0.65)**
Psychiatric diagnoses: No	1 (reference)	1 (reference)	1 (reference)
Psychiatric diagnoses: Yes	**2.49 (2.08 – 2.99)**	**1.73 (1.55 - 1.92)**	**0.83 (0.73 - 0.93)**
**Covariates**			
Past year hospitalization history: before ASI interview			
Not Hospitalized	1 (reference)	1 (reference)	1 (reference)
Hospitalized 1 or more times	**1.31 (1.17 - 1.47)**	**1.77 (1.63 – 1.91)**	**0.68 (0.62 - 0.74)**
Physical Comorbidity			
0 Elixhauser physical comorbidity	1 (reference)	1 (reference)	1 (reference)
1 Elixhauser physical comorbidity	**1.38 (1.23 – 1.56)**	**1.65 (1.51 - 1.81)**	**0.78 (0.70 - 0.86)**
≥2 Elixhauser physical comorbidities	**1.63 (1.45 – 1.83)**	**2.18 (1.99 - 2.39)**	**0.68 (0.62 - 0.76)**
Age Categories	**1.01 (1.00 – 1.02)**	**1.02 (1.01 - 1.03)**	1.00 (0.99-1.01)
50-54 years old	1 (reference)	1 (reference)	1 (reference)
55-59 years	1.03 (0.91 – 1.16)	1.12 (1.03 – 1.23)	0.99 (0.89 – 1.10)
60-64 years	1.05 (0.91– 1.20)	1.14 (1.02 – 1.26)	1.05 (0.93 – 1.19)
65-69 years	1.16 (0.92 – 1.46)	1.40 (1.17 – 1.67)	0.93 (0.76 – 1.13)
70-83 years	1.16 (0.82 – 1.63)	1.42 (1.08 – 1.87)	0.99 (0.71 – 1.37)
Gender			
Man	1 (reference)	1 (reference)	1 (reference)
Woman	**0.84 (0.75 – 0.94)**	0.98 (0.90 - 1.07)	1.07 (0.97 - 1.18)
Country of birth			
Born in Sweden: Swedish parents	1 (reference)	1 (reference)	1 (reference)
Born in other Nordic countries	0.87 (0.74 – 1.03)	1.00 (0.89 - 1.14)	**0.83 (0.71 - 0.96)**
Born outside of Sweden and Nordic Countries	0.91 (0.72 – 1.15)	0.94 (0.79 - 1.13)	1.00 (0.81 - 1.22)
Born in Sweden: Nordic parents	0.87 (0.69 – 1.09)	0.89 (0.75 - 1.06)	1.12 (0.93 - 1.36)
Born in Sweden: non-Nordic parents	0.92 (0.70 – 1.20)	**1.36 (1.10 - 1.68)**	0.92 (0.71 - 1.17)
Marital Status			
Married/cohabiting	1 (reference)	1 (reference)	1 (reference)

Variables	12-months Hospitalized days	Hospital Admission Rate	Time to admission
**Variables**	**IRR (95% CI)**	**IRR (95% CI)**	**IRR (95% CI)**
Separated/widowed	0.98 (0.87 – 1.10)	1.05 (0.96 - 1.15)	0.97 (0.88 - 1.08)
Never married/cohabited	**1.30 (1.06 – 1.59)**	**1.22 (1.05 - 1.42)**	0.99 (0.82 - 1.18)
Usual Employment pattern , past 3 yrs.			
Full/part time employed	1 (reference)	1 (reference)	1 (reference)
Unemployed/irregular/disability	1.06 (0.96 – 1.19)	1.04 (0.96 - 1.13)	0.96 (0.87 - 1.05)
Pension for retired	1.13 (0.89 – 1.43)	1.00 (0.83 - 1.19)	0.97 (0.78 - 1.20)
Study/conscripted/institutionalized	1.13 (0.65 – 1.95)	1.21 (0.83 - 1.76)	0.87 (0.55 – 1. 37)
Education level			
Less than 9 years	1 (reference)	1 (reference)	1 (reference)
Between 9 and 12 years	0.95 (0.82 – 1.09)	**1.15 (1.03 - 1.28)**	1.12 (0. 99 – 1. 27)
Completed 12 years	1.08 (0.90 – 1.30)	**1.22 (1.06 - 1.40)**	1.00 (0.85 - 1.17)
More than 12 years	**1.23 (1.05 – 1.44)**	**1.24 (1.09 - 1.39)**	1.09 (0.95 - 1.25)
Residential town population size			
< 10,000	1 (reference)	1 (reference)	1 (reference)
Between 10,000 and 100,000	1.07 (0.89 – 1.29)	**1.23 (1.07 – 1.41)**	0.98 **(**0.83 - 1.15**)**
> 100,000	1.08 (0.89 – 1.30)	**1.32 (1.15 – 1.52)**	0.96 **(**0.82 - 1.13**)**
Housing condition			
Stable housing	1 (reference)	1 (reference)	1 (reference)
Living with others permanently	**1.22 (1.00 – 1.50)**	1.09 (0.94 - 1.27)	1.07 (0.90 - 1.28)
Housing provided by social services	0.92 (0.76 – 1.12)	0.98 (0.84 - 1.13)	1.18 (1.00 - 1.41)
Unstable housing/Homeless	**1.23 (1.03 – 1.47)**	0.99 (0.87 - 1.14)	1.06 (0.90 - 1.24)
Other e.g., hotel	0.91 (0.70 – 1.19)	1.03 (0.85 - 1.24)	1.21 (0.97 - 1.52)
Criminal justice involvement			
Not sentenced post ASI assessment	1 (reference)	1 (reference)	1 (reference)
Sentenced, but not jailed post ASI assessment	0.99 (0.83 – 1.17)	1.09 (0.99 - 1.21)	1.02 **(**0.90 - 1.15**)**
Jailed 1 or more time post ASI assessment	1.15 (0.89 – 1.51)	1.15 (1.00 - 1.33**)**	1.04 (0.88 - 1.24)
Intercept	5.55 (4.27 – 7.21)	0.00 (0.00 – 0.00)	0.56 (0.44 – 0.70)
/alpha	0.74 (0.69 – 0.80)	0.70 (0.66 – 0.75)	1.15 (1.09 – 1.21)
N	3391	3378	2494

Supplementary Table 5. The RECORD statement checklist of items, extended from the STROBE which should be reported in non-interventional studies using routinely collected data

STROBE=strengthening the reporting of observational studies in epidemiology.

RECORD=reporting of studies conducted using observational routinely collected data.

**Item No**	**STROBE items**	**RECORD items**	**Section (#)**
1	(a) Indicate the study's design with a commonly used term in the title or the abstract.<br>(b) Provide in the abstract an informative and balanced summary of what was done and what was found.	1.1: The type of data used should be specified in the title or abstract. When possible, the name of the databases used should be included.<br><br>1.2: If applicable, the geographical region and timeframe within which the study took place should be reported in the title or abstract.<br><br>1.3: If linkage between databases was conducted for the study, this should be clearly stated in the title or abstract.	Abstract
2	Explain the scientific background and rationale for the investigation being reported.	—	# 1
3	State specific objectives, including any prespecified hypotheses.	—	# 1
4	Present key elements of study design early in the paper.	—	# 2.2
5	Describe the setting, locations, and relevant dates, including periods of recruitment, exposure, follow-up, and data collection.	—	# 2.1
6	(a) Cohort study—give the eligibility criteria, and the sources and methods of selection of participants. Describe methods of follow-up. Case-control study—give the eligibility criteria, and the sources and methods of case ascertainment and control selection. Give the rationale for the choice of cases and controls. Cross sectional study—give the eligibility criteria, and the sources and methods of selection of participants.<br><br>(b) Cohort study—for matched studies, give matching criteria and number of exposed and unexposed. Case-control	6.1: The methods of study population selection (such as codes or algorithms used to identify participants) should be listed in detail. If this is not possible, an explanation should be provided.<br><br>6.2: Any validation studies of the codes or algorithms used to select the population should be referenced. If validation was conducted for this study and not published elsewhere, detailed methods and results should be provided.	[6.1] # 2.1; 2.2<br><br>[6.2] # 2.2; 2.3<br><br>[6.3] # 2.1

Item No	STROBE items	RECORD items	Section (#)
	study—for matched studies, give matching criteria and the number of controls per case.	6.3: If the study involved linkage of databases, consider use of a flow diagram or other graphical display to demonstrate the data linkage process, including the number of individuals with linked data at each stage.	
7	Clearly define all outcomes, exposures, predictors, potential confounders, and effect modifiers. Give diagnostic criteria, if applicable.	7.1: A complete list of codes and algorithms used to classify exposures, outcomes, confounders, and effect modifiers should be provided. If these cannot be reported, an explanation should be provided.	# 2.3
8	For each variable of interest, give sources of data and details of methods of assessment (measurement). Describe comparability of assessment methods if there is more than one group.	—	# 2.3
9	Describe any efforts to address potential sources of bias.	—	# 2.4
10	Explain how the study size was arrived at.	—	-
11	Explain how quantitative variables were handled in the analyses. If applicable, describe which groupings were chosen, and why.	—	# 2.3
12	(a) Describe all statistical methods, including those used to control for confounding.<br>(b) Describe any methods used to examine subgroups and interactions.<br>(c) Explain how missing data were addressed.<br>(d) Cohort study—if applicable, explain how loss to follow-up was addressed. Case-control study—if applicable, explain how matching of cases and controls was addressed. Cross sectional study—if applicable, describe analytical methods taking account of sampling strategy.<br>(e) Describe any sensitivity analyses.	—	# 2.4
12	—	12.1: Authors should describe the extent to which the	[12.1]

Item No	STROBE items	RECORD items	Section (#)
		investigators had access to the database population used to create the study population.<br><br>12.2: Authors should provide information on the data cleaning methods used in the study.	# 2.1<br><br>[12.2]<br><br># 2.3
12	—	12.3: State whether the study included person level, institutional level, or other data linkage across two or more databases. The methods of linkage and methods of linkage quality evaluation should be provided.	# 2.1
13	(a) Report the numbers of individuals at each stage of the study (eg, numbers potentially eligible, examined for eligibility, confirmed eligible, included in the study, completing follow-up, and analysed).<br><br>(b) Give reasons for non-participation at each stage.<br><br>(c) Consider use of a flow diagram.	13.1: Describe in detail the selection of the individuals included in the study (that is, study population selection) including filtering based on data quality, data availability, and linkage. The selection of included individuals can be described in the text or by means of the study flow diagram.	# 2.1; 2.2
14	(a) Give characteristics of study participants (eg, demographic, clinical, social) and information on exposures and potential confounders.<br><br>(b) Indicate the number of participants with missing data for each variable of interest.<br><br>(c) Cohort study—summarise follow-up time (eg, average and total amount).	—	# 2.2;<br><br>Table 1<br><br># 3
15	Cohort study—report numbers of outcome events or summary measures over time. Case-control study—report numbers in each exposure category, or summary measures of exposure. Cross sectional study—report numbers of outcome events or summary measures.	—	# 3
16	(a) Give unadjusted estimates and, if applicable, confounder adjusted estimates and their precision (eg, 95% confidence intervals). Make clear which confounders were adjusted for and why they were included.	—	# 3<br><br>Table 2-3<br><br>Fig 1-2<br><br>Table S4

Item No	STROBE items	RECORD items	Section (#)
	(b) Report category boundaries when continuous variables are categorised.<br>(c) If relevant, consider translating estimates of relative risk into absolute risk for a meaningful time period.		
17	Report other analyses done—eg, analyses of subgroups and interactions, and sensitivity analyses.	—	# 2.4; 3.4; Fig 1-2
18	Summarise key results with reference to study objectives.	—	# 4
19	Discuss limitations of the study, taking into account sources of potential bias or imprecision. Discuss both direction and magnitude of any potential bias.	19.1: Discuss the implications of using data that were not created or collected to answer the specific research question(s). Include discussion of misclassification bias, unmeasured confounding, missing data, and changing eligibility over time, as they pertain to the study being reported.	# 4.1
20	Give a cautious overall interpretation of results considering objectives, limitations, multiplicity of analyses, results from similar studies, and other relevant evidence.	—	# 4
21	Discuss the generalisability (external validity) of the study results.	—	# 4.1
22	Give the source of funding and the role of the funders for the present study and, if applicable, for the original study on which the present article is based.	—	Funding; acknowledge.
22	—	22.1: Authors should provide information on how to access any supplemental information such as the study protocol, raw data, or programming code.	Data access. Statement
